# Internet-based stress recovery intervention FOREST for healthcare staff amid COVID-19 pandemic: study protocol for a randomized controlled trial

**DOI:** 10.1186/s13063-021-05512-1

**Published:** 2021-08-21

**Authors:** Lina Jovarauskaite, Austeja Dumarkaite, Inga Truskauskaite-Kuneviciene, Ieva Jovaisiene, Gerhard Andersson, Evaldas Kazlauskas

**Affiliations:** 1 Center for Psychotraumatology, Institute of Psychology, Vilnius University, M. K. Ciurlionio str. 29, Vilnius, Lithuania; 2 Clinic of Anaesthesiology and Intensive Care, Institute of Clinical Medicine, Faculty of Medicine, Vilnius University, M. K. Ciurlionio Str. 21, LT-03101 Vilnius, Lithuania; 3 Department of Behavioural Sciences and Learning, Department of Biomedical and Clinical Sciences, Linköping University, SE-581 83 Linköping, Sweden; 4 Department of Clinical Neuroscience, Karolinska Institute, Tomtebodavägen 18A, 171 77 Stockholm, Sweden

**Keywords:** Internet-based intervention, Stress recovery, PTSD, Moral injury, Healthcare staff

## Abstract

**Background:**

The demand for care during the COVID-19 pandemic has affected the mental health of healthcare workers (HCWs), thus increasing the need for psychosocial support services. Internet-based interventions have previously been found to reduce occupational stress. The study aims to test the effects of an Internet-based stress recovery intervention—FOREST—among HCWs.

**Methods:**

A randomized controlled trial (RCT) parallel group design with three measurement points will be conducted to assess the efficacy of an Internet-based stress recovery intervention FOREST for nurses. The FOREST intervention is a 6-week Internet-based CBT and mindfulness-based program which comprises of six modules: (1) Introduction, (2) Detachment (relaxation and sleep), (3) Distancing, (4) Mastery (challenge), (5) Control, and (6) Keeping the change alive. We will compare the intervention against a waiting list group at pre-test, post-test, and follow-up. Stress recovery, PTSD, complex PTSD, moral injury, the level of stress, depression, anxiety, and psychological well-being will be measured.

**Discussion:**

The study will contribute to the development of mental healthcare programs for the HCWs. Based on the outcomes of the study, the FOREST intervention can be further developed or offered to healthcare staff as a tool to cope with occupational stress.

**Trial registration:**

ClinicalTrials.gov NCT04817995. Registered on 30 March 2021

## Background

The coronavirus disease 2019 (COVID-19) pandemic has been an enormous challenge for healthcare worldwide, thus putting the mental health of healthcare workers at risk. The increased demand for care during the COVID-19 pandemic has significantly affected healthcare workers’ (HCWs) levels of stress [1, 2], depression [3, 4], and burnout [5–7] and posttraumatic stress disorder [8]. These mental health challenges might be also associated with an experience of moral injury which refers to psychological distress caused by particular actions or absence of them thus violating a person’s moral beliefs [9, 10]. Moral injury is not a mental disorder but it may be related to a negative self-concept and intense negative emotional reactions [10]. Nurses, in particular, are exposed to high psychological distress because they play a crucial role in managing the pandemic-related healthcare crisis [5]. As studies suggest, in the context of the COVID-19 pandemic, nurses may experience emotional exhaustion, depersonalization, and reduced personal accomplishment [6] among other mental health issues.

Despite the obvious increase in demand for psychosocial support during the pandemic, access to tailored psychological services focused on reducing occupational stress in nurses and other medical personnel is limited. Additionally, healthcare workers’ unwillingness to seek psychological help also contributes to this [11]. Furthermore, public health measures and the closure of healthcare services during the pandemic restrict access to traditional mental health services. Internet-based interventions have been found to be effective for a range of mental health conditions [12], including life-stressor-related adjustment disorders [13] as well as burnout among HCWs [14]. Moreover, especially during the COVID-19 pandemic, online therapies are particularly relevant for HCWs because of their flexibility, access to a large-scale number of medical staff, and the possibility to provide psychosocial care for HCWs from isolated regions [15].

The current study aims to test the efficacy of stress recovery intervention FOREST among HCWs, in particular, nurses with high levels of stress in the context of the COVID-19 pandemic. The FOREST intervention was developed based on the theoretical framework of stress recovery [16] which emphasizes the importance of stress self-awareness, life-work balance, and self-care. The program was specifically developed to address the needs of HCWs amid the COVID-19 pandemic and was designed as a CBT and mindfulness-based Internet-delivered intervention to reduce barriers to accessing the intervention.

The primary objective of the trial is:

1. To evaluate the efficacy of the Internet-based for stress recovery (FOREST) intervention in improving stress recovery among nurses in comparison to a waiting list control group in the context of the COVID-19 pandemic

The secondary objectives are:

2. To assess the effect of the FOREST intervention on posttraumatic stress disorder (PTSD) as well as complex posttraumatic stress disorder (CPTSD) symptoms
3. To investigate the effect of the FOREST intervention on moral injury
4. To evaluate the effect of the FOREST program on the perceived level of stress
5. To assess the effect of the FOREST program on depression and anxiety
6. To evaluate the effect of the FOREST program on psychological well-being

## Methods

### Study design and setting

A randomized controlled trial (RCT) parallel groups waiting list design with three measurement points will be used to assess the efficacy of an Internet-based stress recovery intervention FOREST for HCWs, i.e., nurses (superiority trial). We aim to recruit 600 participants in Lithuania and, based on previous e-health studies [17], we expect a dropout of 30%. This will generate sufficient statistical power to detect differences between the groups on the primary outcome measure of stress recovery given a significance level of .05 and a power of 80% [18]. Participants will be randomly allocated to the intervention or a waiting list control group with an allocation ratio of 1:1. Participants allocated to the intervention condition will receive the intervention immediately after randomization, and participants in the waiting list condition will be offered the same intervention 6 months later. The intervention will last for 6 weeks. The pre-test, the post-test, and the 3-month follow-up will be carried out at the same time in both study groups. We will compare stress recovery, PTSD and CPTSD symptoms, moral injury, perceived stress, anxiety, depression, and psychological well-being in nurses who participate in the FOREST intervention vs. those on the waiting list. All study measures will be self-reported and administrated via a secure web application [19]. All participants included in the study will get personalized login data on the first day of using the program. Once participants of the study create a secure password, they will be able to log into the platform where they will have access to the content of the intervention, as well as communication with a psychologist. All the content on the platform is private and protected by end-to-end encryption and participants use secure login for each connection to the platform.

**Fig. 1:**
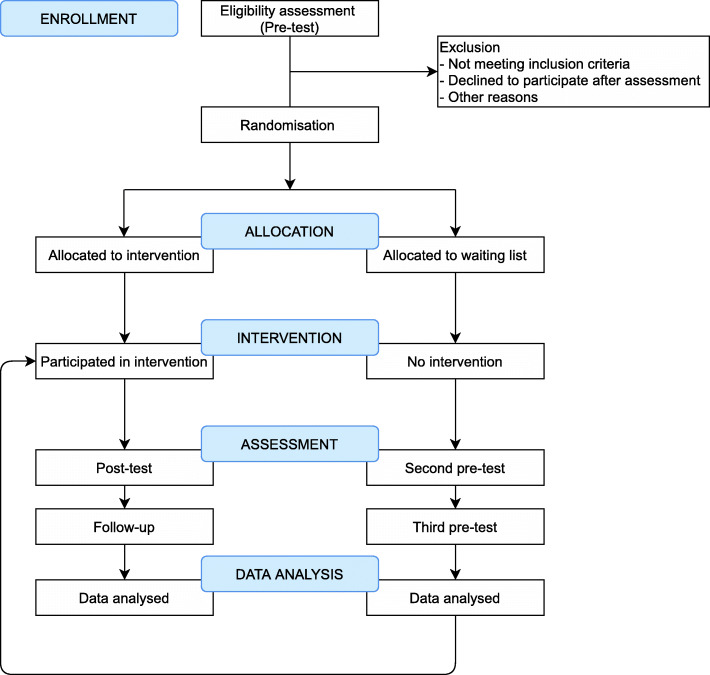
Flowchart of the intervention

This study protocol is following the Standard Protocol Items: Recommendations for Interventional Trials (SPIRIT) 2013 Checklist [20]. The information regarding enrolment, intervention, and assessments in the trial are presented in Table 1. In addition, the details of the rationale of the study are shown in the flowchart (Fig. 1). The ethics approval for the trial was obtained from the Institutional Psychological Research Ethics Committee of Vilnius University (2021-03-22/61). All study participants will be also asked to give a written consent online in order to participate in the study.

**Table 1:** Enrolment, interventions, and assessments of the FOREST

	Enrolment	Allocation	Post-allocation				Close-out
Timepoint	*t*_*1*_		*t*_*2*_	*t*_*3*_	*t*_*4*_	*t*_*5*_	
Enrolment							
Informed consent	X						
Assessment	X						
Eligibility screen		X					
Randomization		X					
Final allocation		X					
Interventions							
Intervention group		X	X				
Waiting list control group				X	X		
Assessments							
Recovery experiences	X		X	X	X	X	
PTSD and CPTSD	X		X	X	X	X	
Moral injury	X		X	X	X	X	
Stress	X		X	X	X	X	
Depression	X		X	X	X	X	
Anxiety	X		X	X	X	X	
Psychological well-being	X		X	X	X	X	
Post-assessment interviews							X

### Participants and inclusion/exclusion criteria

The study will enroll self-referred participants after dissemination of invitations to professional HCWs’ social networks (e.g., social networks of nurses in different regions of Lithuania), healthcare institutions (e.g., primary healthcare centers, hospitals), and press release to national media. The healthcare institutions and administrative staff of HCWs’ social networks will be asked to share the invitation with nurses. No monetary compensation will be offered for the dissemination of study advertisements. Licensed nurses working in the healthcare system throughout the country will be enrolled in the study. To be eligible to participate in the study, applicants must provide written informed consent online, are required to complete a baseline assessment prior to randomization, and meet all of the following criteria: to be at least 18 years old, to comprehend Lithuanian to the degree that one understands the content and instructions of the study, and to have a computer, tablet, smartphone, or similar device with access to the Internet. Applicants meeting any of the following criteria will be excluded from participation in this study: acute psychiatric crisis, high suicide risk, alcohol/drug addiction, and interpersonal violence. For the secondary eligibility check, before the randomization, all the participants will be contacted by phone call for a brief interview to clarify their eligibility for the study.

### Randomization

Randomization will be conducted by the researcher not associated with the study team using the random number calculation procedure using www.random.org. In the randomization process, eligible participants’ IDs will be used to allocate HCWs to the intervention or the waiting list groups. All study participants will be informed whether they are allocated to the intervention or the control group after completing the study measures at the baseline measurement point. Furthermore, participants allocated to the waiting list will be asked to fill in the measures at the same measurement points as the intervention group and will be invited to participate in the FOREST intervention after that.

### Intervention condition

The FOREST intervention is a modification of the intervention for distressed employees which has been adjusted to the specific needs of the HCWs, i.e., nurses, meaning that the current intervention is more focused on the specific HCWs’ profession-related stressors and mental health issues. The intervention is developed as a guided program with active individualized messaging-based feedback from psychologists following the completed tasks of the intervention as well as psychologist’s support on-demand as a response to the written messages initiated by the intervention participants [21]. In addition, all intervention participants will be contacted by a phone call in the middle of the intervention (after 3 weeks) and at the end of the intervention (after 6 weeks) by their psychologist for a brief interview regarding the usage of the program. The FOREST will be delivered through a secure online platform [19], which has been used in various previous studies and has been translated into Lithuanian.

**Fig. 2:**
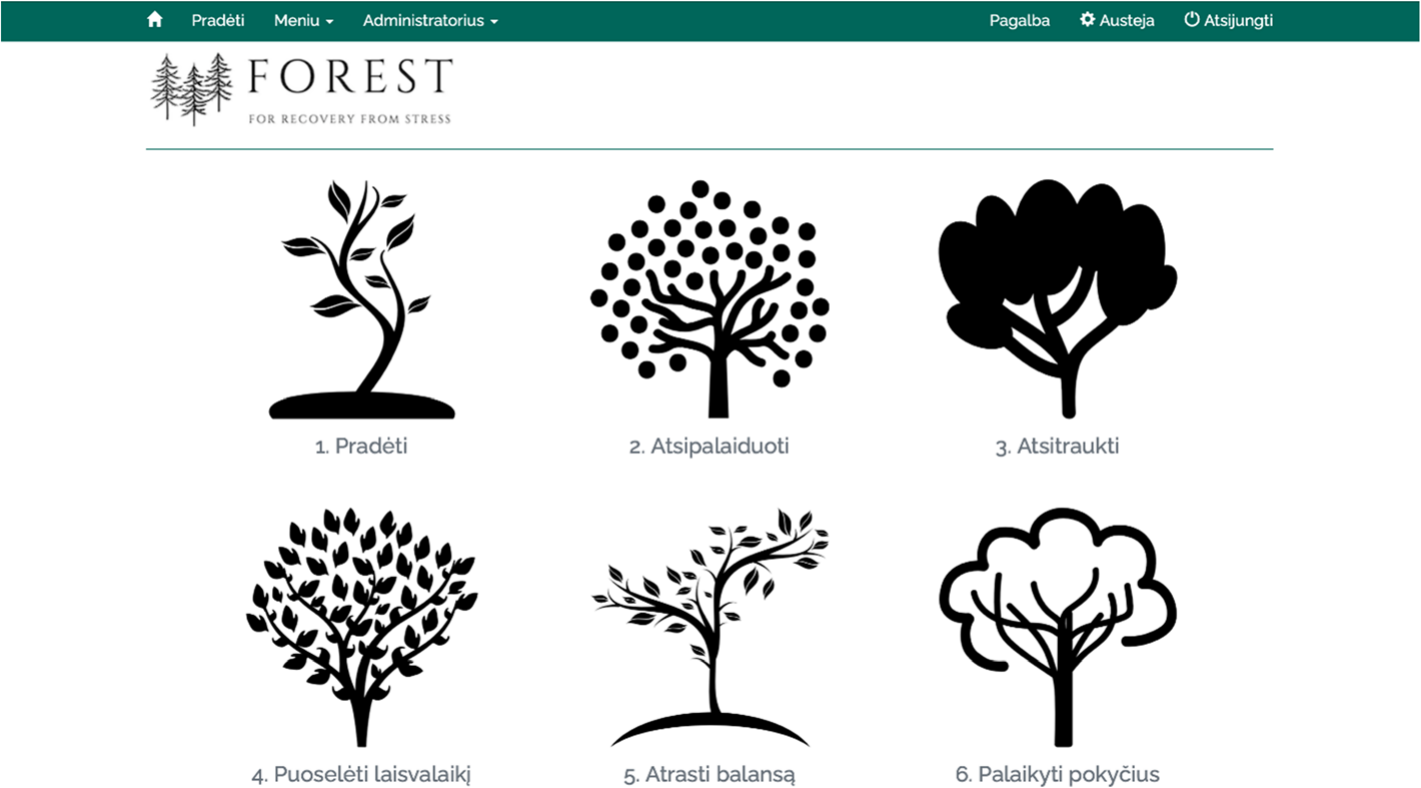
The interface of the FOREST intervention

The content of the FOREST intervention has been developed by the team of clinical psychologists and it is based on cognitive behavior therapy (CBT) principles and mindfulness. The FOREST intervention comprises six modules (the interface of the FOREST is presented in Fig. 2): (1) Introduction, (2) Detachment (relaxation and sleep), (3) Distancing, (4) Mastery (challenge), (5) Control, and (6) Keeping the change alive. The content of the FOREST intervention is presented in Table 2. Each of the six intervention modules consists of psychoeducation (written texts as well as video recordings), two or three exercises for a participant, and a reminder of the opportunity to contact the therapist. Also, tasks for participants will be provided in several formats, namely, listening for audio records, or in the form of written responses to module-related questions. All the audio records will be available for download. Moreover, study participants will be able to choose the intensity of the program according to their personal needs but will be encouraged to complete the exercises to reach the best results on weekly basis. Access to a new module will be provided every week on the same weekday over the 6 weeks. Once accessible, modules will remain available throughout the intervention.

**Table 2:** The content of the six modules of the FOREST intervention

Module	Aim and content of the module	Module exercises
1. Introduction	Introduction to the intervention (aims, benefits, instructions of usage) Psychoeducation about stress and burnout, its benefits and harms Brief psychoeducation about stress recovery: detachment (relaxation and sleep), distancing, mastery (challenge), control	1. Selecting stressors from the list and naming three most prominent work- and personal life-related stressors 2. Selecting burnout symptoms from the list 3. Short breathing exercise (an audio record)
2. Detachment (relaxation and sleep)	Psychoeducation about body relaxation and its benefits Psychoeducation about sleep, its benefits, and harms of prolonged sleep difficulties	4. Body relaxation (an audio record), evaluation of stress before and after the exercise, naming associations that emerged during relaxation 5. Sleeping relaxation (an audio record)
3. Distancing	Psychoeducation about distancing (both physically and mentally) and its benefits in dealing with work and personal life challenges Psychoeducation about intrusive thoughts	6. Naming three activities that help to keep the distance from work 7. Awareness of thoughts (an audio record) and naming emotions briefly after it 8. Mindful walking (an audio record)
4. Mastery (challenge)	Psychoeducation about mastery in day-to-day activities and its benefits Psychoeducation about physical exercise, its benefits, and relations to stress recovery	9. Selecting activities from the list (both active and less active) or filling in one’s own 10. Short pause (an audio record) 11. Short body stretching (video record)
5. Control	Psychoeducation about control over one’s life and its benefits Psychoeducation about the importance of self-care and control over one’s working day structure and its benefits	12. Naming unnecessary and bothering activities, changing them to pleasant and relaxing activities 13. Reflecting on daily goals (an audio record)
6. Keeping the change alive	Summarizing the program and main aspects of the provided information Encouraging to further practice the intervention exercises after completion of the intervention	14. Brief relaxation exercise (an audio record)

Five clinical psychologists and five master students in the clinical psychology program will be involved as psychologists in the FOREST intervention. They will all receive special training according to the guidelines specifically developed for the study. Weekly supervision meetings will be scheduled, and supervision will also be provided on request. The role of each psychologist will be to give feedback to study participants regarding the use of the intervention and their psychological well-being or answer their other questions. Psychologists’ feedback will be largely structured and standardized; nevertheless, personalized responses will be encouraged to correspond to the particular case. Study participants and psychologists will communicate within a secure platform [19]. Psychologists will be asked to provide feedback to participants’ comments in 24 h.

Psychologists will respond to signs of deterioration of mental health noticed from the communication with the intervention participant, e.g., suicide threat, by referring to other mental health services. Study participants in need will be provided with the information regarding the mental health services in the community.

### Data management

Outcome data will be collected at the three time points using the secure online platform [19]. In addition, data on the usability of the program, such as number of logins, completed exercises, or texts to a psychologist, will be collected within the same platform. All participants will be given an anonymous identifying number. Access to data will be restricted to researchers directly involved in the study only and via a secure login with two-step authentication. All data collected will be stored and managed according to current national regulations of personal data management. All participants will be informed that the data provided will be treated confidentially and will be made aware that in published reports it will not be possible to identify any individual or attribute any information to them.

The collected data will be exported and inputted into SPSS files for analyses and saved on secure university storage. The data file with personal information will be accessible for the researchers directly involved in the study only. Access to the data file with an anonymous identifying number may be provided for the senior and/or junior data analyst as well as made publicly available as a part of the open research data policy required by the journal as a condition for publication of research outputs.

Dropout or premature termination from the study at any point after randomization will be recorded. Participants will be able to choose to withdraw from the study at any point and may ask that previously collected data not be used. Unless a participant has withdrawn consent to participation, repeated attempts will be made to contact participants who will not complete the outcome assessments. All participants will be asked to complete the study measures at each point of measurement, regardless of protocol adherence or any previously uncompleted measures.

### Primary outcome

#### Stress recovery

The Recovery Experiences Questionnaire (REQ) [22] will be used to evaluate the changes in self-reported recovery of stress in HCWs. All 16 items of the REQ are ranked on a 5-point Likert scale ranging from 1 (= totally disagree) to 5 (= totally agree). The scores of the total REQ scale range from 16 to 80. A higher score indicates a more pronounced recovery of stress. Good psychometric properties have been reported previously for the total REQ (Cronbach *α* = 0.92) as well as for subscales, i.e., psychological detachment (Cronbach *α* = 0.88), relaxation (Cronbach *α* = 0.86), mastery (Cronbach *α* = 0.84), and control (Cronbach *α* = 0.89) [23].

### Secondary outcomes

#### Posttraumatic stress disorder

The International Trauma Questionnaire (ITQ) [24, 25] will be used to measure self-reported symptoms of posttraumatic stress disorder (PTSD) and complex posttraumatic stress disorder (CPTSD). The ITQ is a widely used measure for ICD-11 PTSD and CPTSD. All 18 ITQ items are evaluated on a 5-point Likert scale ranging from 0 (= not at all) to 4 (= extremely). The scores of the total PTSD and DSO range from 0 to 24. A higher score indicates more severe symptoms of PTSD or CPTSD. Additionally, we will apply a diagnostic algorithm for the diagnosis of PTSD and CPTSD based on the clinical significance of the symptoms and functional impairment [24]. Good psychometric properties were reported for both PTSD as well as DSO subscales (Cronbach *α* ≥ 0.79) [24].

#### Moral injury

The Moral Injury Outcome Scale (MIOS) [26] will be used to measure self-reported rates of moral injury. The MIOS is comprised of 14 items. All the MIOS items are ranked on a 5-point Likert scale ranging from 0 (= strongly disagree) to 4 (= strongly agree). The scores of the total MIOS range from 0 to 56. A higher score indicates a more pronounced moral injury. The MIOS is a state-of-the-art measurement instrument for moral injury and ongoing studies are currently being implemented regarding the investigation of the psychometric properties of the MIOS in HCWs.

#### Stress

The Perceived Stress Scale (PSS-4) [27] will be used to assess the level of perceived stress. The PSS-4 is a brief scale comprising of 4 items ranked on a 5-point Likert scale ranging from 0 (= never) to 4 (= very often). The scores of the total PSS-4 range from 0 to 16. A higher score indicates more pronounced perceived stress. Good psychometric properties were reported previously for the PSS-4 (Cronbach *α* = 0.75) [28].

#### Depression and anxiety

The Patient Health Questionnaire-4 (PHQ-4) [29] will be used to measure depression and anxiety symptoms. The PHQ-4 is a self-reported scale comprising of 4 items. All the items are ranked on a 4-point Likert scale ranging from 0 (= not at all) to 3 (= nearly every day). The scores of the total PHQ-4 vary from 0 to 12. A higher score indicates more pronounced depression and anxiety symptoms. Good psychometric properties were reported previously for the total scale (Cronbach *α* = 0.86) in the sample of HCWs [30].

#### Psychological well-being

The World Health Organization Well-being Index (WHO-5) [31] will be used to measure psychological well-being. The WHO-5 index is a self-report scale comprising of 5 items. All the items are ranked on a 6-point Likert scale ranging from 0 (= at no time) to 5 (= all the time). The scores of the total WHO-5 vary from 0 to 25. A higher score indicates higher psychological well-being. Good psychometric properties were found in a previous study of the Lithuanian version of WHO-5 (Cronbach *α* = 0.92) in the sample of HCWs [1].

#### Other measures

Study participants will be asked to evaluate the usability of the FOREST intervention by ranking how useful (1 = not useful at all to 5 = very useful), satisfactory (1 = I did not like it at all to 5 = I liked it a lot), and easy to use (1 = it was not easy at all to 5 = it was very easy) the intervention has been. They will also be asked to report their subjective impression regarding the improvement of mental well-being (1 = worsened a lot to 5 = improved a lot), general understanding of oneself, and one’s well-being (1 = not at all to 5 = definitely improved) and recommending the program to others (1 = not at all to 5 = definitely would recommend).

### Statistical analyses

As the study aims to capture the possible change in primary and secondary outcomes in the intervention group, in comparison to the control group, a series of mixed multivariate repeated-measures ANOVAs with time (pre-test, post-test, and follow-up) as a within-subject factor and group (intervention vs. control) as a between-subject factor will be performed. Continuous data aggregation will be used; the change from baseline of the outcome measures sum scores will be recorded. Additionally, we will calculate both within- and between-group effect sizes. The between-group effect sizes will be calculated by using the mean difference from pre-test to post-test (for the short-term effect) and from pre-test to follow-up (for the long-term effect) in the intervention and control groups and the standard deviations of each group at the pre-test [32]. The within-group effect sizes will be calculated by using the means in each group at pre- and post-test/follow-up and standard deviations at each measurement point. The magnitude of the effect expressed in *d* will be interpreted as follows: 0.50 = medium effect and ≥ 0.80 = large effect [18]. The data will be analyzed by using the intention-to-treat principle [33]. The missingness of the data will be treated by using the multiple imputation method [34].

## Discussion

This study will be among the first which will evaluate the effects of the Internet-based psychosocial intervention on stress levels and mental health of nurses in the context of the COVID-19 pandemic. Work overloads and long working hours contribute to high levels of stress in nurses [3] and new solutions are needed for psychosocial care. Nurses are particularly burdened and are at risk for burnout [5]; thus, the study is targeted to nurses.

The current intervention is developed to address the needs of the HCWs by using digital technologies which increases the usability of the program by providing flexibility while accessing the intervention. Access to intervention via their digital device reduces barriers of help-seeking, as participants of the trial can access the program with high flexibility without the need for appointments with mental health professionals which is particularly important for healthcare staff who often have long working hours and busy schedules. Media resources developed for this intervention, such as audio and video recordings, will make the intervention usable and attractive for the users.

The current study will contribute to the development of mental healthcare programs for HCWs. Based on the outcomes of the study, the FOREST intervention can be further developed or offered to the healthcare staff as a tool to cope with work-related stress and increase well-being if outcomes will show positive effects of the intervention on the study participants. Specifically, the study will fill the gap in the scientific knowledge regarding the short- and long-term effects of Internet-based stress recovery intervention on the mental health of HCWs. Additionally, the study will provide evidence of the impact of Internet-based stress recovery intervention on a moral injury which is an especially relevant experience for HCWs [10].

The study contains several potential risks. The target group of the study has high working loads during the COVID-19 pandemic, thus, leading to potential issues in terms of the study participants’ recruitment, data collection, and adherence. Data collection and compliance with study procedure risks will be managed via communication with a psychologist on the platform, periodic reminders to enter the new intervention session, and phone calls aimed to receive feedback about the study.

## Trial status

Recruitment of participants began 01/04/2021 and will continue to 30/06/2021.

## Data Availability

The data file will not be publicly available. An anonymous copy of the data file of this study will be available from the corresponding author upon reasonable request.

## Abbreviations

CPTSD: Complex posttraumatic stress disorder
COVID-19: The coronavirus disease 2019
HCW: Healthcare worker
MIOS: Moral Injury Outcome Scale
PHQ-4: Patient Health Questionnaire
PSS-4: Perceived Stress Scale
PTSD: Posttraumatic stress disorder
REQ: Recovery Experiences Questionnaire
RCT: Randomized controlled trial
WHO-5: World Health Organization Well-being Index
